# Challenging Oil Bioremediation at Deep-Sea Hydrostatic Pressure

**DOI:** 10.3389/fmicb.2016.01203

**Published:** 2016-08-03

**Authors:** Alberto Scoma, Michail M. Yakimov, Nico Boon

**Affiliations:** ^1^Center of Microbial Ecology and Technology, University of GentGent, Belgium; ^2^Institute for Coastal Marine Environment – National Council of ResearchMessina, Italy; ^3^Immanuel Kant Baltic Federal UniversityKaliningrad, Russia

**Keywords:** marine snow, dispersants, beta-oxidation, HMW, PAH, *Alcanivorax*, *Thalassolituus*, burn residue

## Abstract

The Deepwater Horizon accident has brought oil contamination of deep-sea environments to worldwide attention. The risk for new deep-sea spills is not expected to decrease in the future, as political pressure mounts to access deep-water fossil reserves, and poorly tested technologies are used to access oil. This also applies to the response to oil-contamination events, with bioremediation the only (bio)technology presently available to combat deep-sea spills. Many questions about the fate of petroleum-hydrocarbons within deep-sea environments remain unanswered, as well as the main constraints limiting bioremediation under increased hydrostatic pressures and low temperatures. The microbial pathways fueling oil bioassimilation are unclear, and the mild upregulation observed for beta-oxidation-related genes in both water and sediments contrasts with the high amount of alkanes present in the spilled oil. The fate of solid alkanes (tar), hydrocarbon degradation rates and the reason why the most predominant hydrocarbonoclastic genera were not enriched at deep-sea despite being present at hydrocarbon seeps at the Gulf of Mexico have been largely overlooked. This mini-review aims at highlighting the missing information in the field, proposing a holistic approach where *in situ* and *ex situ* studies are integrated to reveal the principal mechanisms accounting for deep-sea oil bioremediation.

## Deep-Sea Oil Contamination

Contamination of deep-sea environments with petroleum following accidental spills represents a relatively emerging topic, which received worldwide attention after the Deepwater Horizon (DWH) accident at the Gulf of Mexico in April 2010 (Joint Analysis Group [JAG], 2010) when more than 500’000 tons of crude oil (+24% including gas; Reddy et al., 2012) were discharged at ∼1500 m below surface level (bsl; U.S. Geological Survey, 2010). As political reasons will keep pushing for deep-sea oil extraction, use of poorly tested technologies is not expected to decrease the risk of future accidents (Jernelov, 2010; Thibodeaux et al., 2011).

**FIGURE 1:**
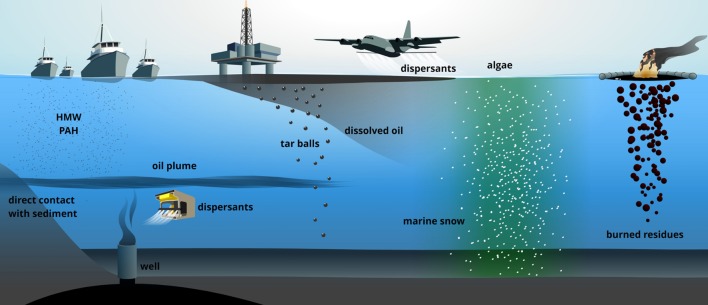
**The many ways through which accidentally-spilled petroleum-hydrocarbons reach the seafloor and deep-sea.** HMW PAH, high molecular weight polycyclic aromatic hydrocarbons.

Spilled oil reaches the deep sea through numerous ways. Surface-water spills form thin layers which partially dissolve, emulsify and diffuse through the water column (Tkalich et al., 2003), or sink due to the formation of heavier particles (tar) (Parinos et al., 2013). Dispersants enhance oil solubility in water. Extensive injections of the dispersant COREXIT at deep sea (about 3 × 10^6^ L) during the DWH contributed to the formation of a large oil plume at 1000–1300 m bsl (Camilli et al., 2010) preventing petroleum-hydrocarbons from reaching the surface (Kujawinski et al., 2011). Direct contact of the plume with the continental slope was partially responsible for contamination of deep-sea sediments (Romero et al., 2015). Another vector for sinking oil is the “so-called” marine snow. Oil contamination enhances phytoplankton production of exopolysaccharides (EPSs, Patton et al., 1981), whose amphiphilic nature favors hydrophobic-hydrophobic interactions with oil to form particles including microbial biomass that sink downward (Passow et al., 2012). This phenomenon represented the main cause for oil transfer to the seafloor during the DWH (Federal Interagency Solution Group, 2010; Ziervogel et al., 2014; Romero et al., 2015). *In situ* oil burning, one of the most widely applied strategies for oil-pollution control, is known to cause seafloor contamination (Wang et al., 1999; Federal Interagency Solution Group, 2010). Following mechanical oil recovery through skimming, the unrecoverable oil fraction on the surface is gathered within small areas for controlled burning, which generates denser mixtures of the less volatile fraction of the oil (resins, asphaltenes; Buist et al., 1997; Jézéquel et al., 2014). Finally, geochemical data on the increased heavy-molecular-weight polyaromatic hydrocarbons (PAHs) fraction in DWH deep-sea sediments indicated that diesel exhaust from the 6000 vessels conducting safety operations cannot be excluded as contamination factor (Romero et al., 2015; **Figure 1**).

There has been little to no effort in assessing the magnitude of deep-sea oil contamination worldwide. Between 3200 and 8000 km^2^ of deep seafloor were impacted with up to 14% of the DWH spilled-oil (Chanton et al., 2014), although ∼22% could not be traced (Ramseur, 2010). The impact on deep-sea life was striking. Deep-sea sediments were classified as low to moderately polluted (Romero et al., 2015); pore-water from 1000 to 1400 m bsl exerted high toxicity levels and DNA mutagenesis (Paul et al., 2013); primary production and carbon export to the deep-sea was reduced (Prouty et al., 2016); in sea-food, concentration of certain petroleum-hydrocarbons was 1000 times above the threshold for human consumption (Sammarco et al., 2013).

Hydrocarbons enter deep-sea areas also through several geochemical routes (oil seeps, hydrothermal vents, gas hydrates, asphalt volcanoes; Jørgensen and Boetius, 2007). Several microorganisms proficiently use oil as an energy/carbon source preventing its accumulation into marine environments (Head et al., 2006). Natural niches characterized by fossil hydrocarbons determine microbial community structures featured by unique biochemical equilibria, which form over a time-span of centuries (Jørgensen and Boetius, 2007). Conversely, anthropogenically oil-affected sites are non-adapted environments where overabundant carbon loads are discharged within weeks/months. The enrichment of oil-degrading taxa jump-starting bioremediation coincides with a net loss of biodiversity (Kleindienst et al., 2015), which can hardly be recovered until excess oil has been depleted. The oligotrophic nature of marine environments (Garrison, 2015) limits oil bioassimilation, which is further impaired at the deep sea by low temperatures, O_2_ availability, and hydrostatic pressure (HP).

## Review Objective

Lack of efficient oil recovery technologies at deep sea implies that bioremediation represents the only mean to combat contamination (Lu et al., 2012). The DWH spill was investigated through *in situ* studies employing next-generation sequencing techniques, which were partially backed-up by *ex situ* experiments. Despite supplying unprecedented information, both approaches failed to describe the exact metabolic routes and constraints in deep-sea bioremediation. *In situ* studies using molecular techniques could only provide information on potential activities while, with no exception, *ex situ* studies neglected the impact of one of the major drivers for biodiversity in marine environments, i.e., HP (Ghiglione et al., 2012). While previous overviews focused on microbial succession (Kimes et al., 2014), marine snow formation (Joye et al., 2014) and hydrocarbon fate (King et al., 2015) following the DWH, this mini-review aims at highlighting the open questions concerning the physiology of oil bioremediation at deep-sea HP conditions.

## *In Situ* Molecular Studies: Deep-Sea Plume

Upon injection, DWH spilled-oil was composed of 74, 16, and 10% saturated, aromatic and polar hydrocarbons, respectively; gas represented 24% of the spill, while oil comprised 76% (alkanes being 32% of the total; **Table 1**). Fractionation due to physicochemical factors (and dispersants application; Kujawinski et al., 2011) resulted into different petroleum mixtures affecting water and sediment. The oil plume was mainly composed of gaseous and monoaromatic compounds (**Table 1**). A hydrocarbon-dependent microbial community restructuring was proposed for the plume. Following a first enrichment in *Oceanospirillales* and *Pseudomonas* (May 2010), the relative increase in aromatic hydrocarbons as compared to aliphatic and cycloalkanes following partial cap closure (June 4, 2010) coincided with a general shift in dominance to *Colwellia, Cycloclasticus, Pseudoalteromonas* and methylotrophs lasting until mid-August 2010 (Hazen et al., 2010; Valentine et al., 2010; Kessler et al., 2011; Lu et al., 2012; Mason et al., 2012; Dubinsky et al., 2013; Rivers et al., 2013; Kleindienst et al., 2015). Before partial closure of the well, metagenomic (Hazen et al., 2010; Lu et al., 2012; Mason et al., 2012) and metatranscriptomic (Mason et al., 2012; Rivers et al., 2013) analyses evidenced an upregulation of genes related to hydrocarbon degradation, although a consensus could not be reached. Upregulation of genes or pathways related to monoaromatics or PAH degradation was observed in all studies to different extents (Hazen et al., 2010; Lu et al., 2012; Mason et al., 2012; Rivers et al., 2013). Mason et al. (2012) found a higher level of gene and transcript reads related to the degradation of *n*-alkanes rather than aromatics, contrary to Hazen et al. (2010). The latter would be in contrast with data from the same group indicating that *n*-alkane and cycloalkane concentrations correlated with the enriched communities before partial closure (Dubinsky et al., 2013). Similarly, the upregulation of the cyclohexanone degradation pathway as detected in Mason et al. (2012) was negligible in Hazen et al. (2010). Upregulation of alkane-1 mono-oxygenases responsible for *n*-alkane activation was detected in all studies (Hazen et al., 2010; Lu et al., 2012; Mason et al., 2012; Rivers et al., 2013). Following activation, *n*-alkanes should enter beta-oxidation (Rojo, 2009), but genes related to this pathway were only partially upregulated in Mason et al. (2012) and Rivers et al. (2013). Upregulation of anaerobic hydrocarbon degradation genes (Lu et al., 2012) was consistent with that for nitrate reduction (Rivers et al., 2013), although O_2_ levels were marginally affected at that time (Camilli et al., 2010; Hazen et al., 2010). As 16S rRNA gene signatures persisted long after the plume had dissipated (Joye et al., 2014), such controversies concerning structure-function relationships highlight the need for more data integration (Widder et al., 2016).

**Table 1:** Detected oil fractions in water and sediment deep-sea samples after the Deepwater Horizon spill.

Deepwater Horizon spilled-oil composition at origin (15 MPa, 5°C)			Oil		Oil plume							Sediments				
					
			x10^9^ g	%												
		
2010					2010								2010		2011	
		
Sampling period	June 21				May 9–16	May 25–June 6	May 25–June 2	May 25–June 2	June 11–21	June 19–28	August 18–October 4	September	September 16–October 20	December–February	May	November 16
	Methane		100	14.3		+			+	+	+					
	Ethane		19	2.7		+			+		+					
	Propane		18	2.6		+			+		+					
Gas	Isobutane		4.7	0.7		+										
	*n-butane*		10	1.4		+										
	Isopentane		5.6	0.8												
	*n-pentane*		7.3	1		+										

	*n-alkanes*		81	11.6			+	+					+		+	+
	Branched alkanes		140	20.1									+			
	Cycloalkanes		84	12.1									+			
Oil	Alkylbenzenes and indenes		48	6.9											+	
	BTEX		19.2	2.8			+	+		+					+	
	PAH		21	3	+								+	+	+	
	Unresolved/not defined		139.6	20								+		+	+	
	Polar		54	7.7												

	Total gas		165	24												
Oil and gas	Total oil		533	76												
	Total spill		697	100												

Reference		Reddy et al., 2012			Diercks et al., 2010	Joye et al., 2011	Hazen et al., 2010	Lu et al., 2012	Valentine et al., 2010	Camilli et al., 2010	Kessler et al., 2011	Sammarco et al., 2013	Mason et al., 2014	Romero et al., 2015	Liu et al., 2012	Yergeau et al., 2015


While a microbial and molecular response to oil appears evident, the actual metabolic routes following hydrocarbons uptake are not. Lack of significant O_2_ respiration in the plume early in the spill contrasts with the enhanced cell number (Hazen et al., 2010; Mason et al., 2012; Kleindienst et al., 2015). Sustained aerobic biodegradation would be expected to result in increased CO_2_ production and decreased pH, but none of these phenomena were reported. The ease of hydrocarbon degradation would suggest the plume to be enriched in *n*-alkane degraders as proposed for gaseous compounds (Valentine et al., 2010) and the *Oceanospirillales* group found by Hazen et al. (2010), but the preferential molecular response to aromatics brings this hypothesis into question. The fate of *n*-alkanes (>C_6_) and the lack of a strong beta-oxidation upregulation in the plume remain unexplained.

Persistent O_2_ anomalies were observed following well closure (July 15, 2010). Their strong intensity was used to track the oil plume moving southwestward (Du and Kessler, 2012) and associated with heterotrophic degradation of high-molecular-weight organics rather than oil (Dubinsky et al., 2013)- possibly due to marine snow formation after the spill (Ziervogel et al., 2014)- or with aerobic degradation of gaseous hydrocarbons (Kessler et al., 2011). The latter would be consistent with enriched methylotrophs at that time (Kessler et al., 2011; Redmond and Valentine, 2012; Dubinsky et al., 2013; Kleindienst et al., 2015). However, actual CH_4_ respiration rates were inconsistent throughout the spill (Crespo-Medina et al., 2014) and CH_4_ mono-oxygenase upregulation before May–June 2010 is still under debate (Joye et al., 2014; Kimes et al., 2014; King et al., 2015). Joye et al. (2014) suggest that some presently unknown factor hampered CH_4_ respiration after mid-June. As it stands, venting to the atmosphere could not be excluded (Rivers et al., 2013).

## *In Situ* Molecular Studies: Deep-Sea Sediments

Deepwater Horizon sediments were investigated through metagenomic analysis of seafloor (Mason et al., 2014) and sub-seafloor (Kimes et al., 2013) samples in September–October 2010. Superficial samples (0–1 cm) within 5 km of the wellhead were the most impacted, and were enriched in uncultured *Gammaproteobacteria* and *Colwellia*-related organisms similar to the ones in the plume, and in uncultured *Rhodobacteraceae* (Mason et al., 2014). Highly impacted sediments showed increased levels of monoaromatic degradation genes, no relation with PAH and O_2_ respiration, thus partly resembling results obtained for plume samples in May-June (Hazen et al., 2010; Lu et al., 2012; Rivers et al., 2013). Sub-seafloor samples (1–3 cm deep) close to the wellhead (0.5 and 6 km, Kimes et al., 2013) were enriched in several *Deltaproteobacteria*, although *Alpha*- and *Gammaproteobacteria* remained predominant (Kimes et al., 2013). Enhanced expression of the *bssA* gene was consistent with increased benzylsuccinate levels formed *via* the fumarate pathway. Conversely, no alkylsuccinate or alkylmalonate metabolites related to alkane degradation were found despite the corresponding *assA* gene being upregulated and alkanes up to *n*-docosane being detected (Kimes et al., 2013), reflecting the uncertain fate of *n*-alkanes (>C_6_) in the plume. Consistently, genes related to fatty acids metabolism were poorly expressed in seafloor sediments close to the wellhead (Kimes et al., 2013) and expression levels were comparable to pre-spill samples (Kimes et al., 2013). One year later, *n*-alkanes (C_8_–C_38_) were 10–1000 times more concentrated than PAH in the sediments (Liu et al., 2012), suggesting that the degradation of PAH was faster than that of *n*-alkanes (Yergeau et al., 2015; **Table 1**).

As for the plume, CH_4_ respiration rates in sediments are uncertain. After 1 year, the upper (0–2 cm) sediments located 2–6 km from the wellhead were populated by *Actinobacteria*, *Firmicutes*, *Chloroflexi* and several type I methylotrophs (Liu and Liu, 2013). Provided that CH_4_ levels in the plume were low in October 2010 (Kessler et al., 2011; Crespo-Medina et al., 2014), it appears unlikely that plume-related CH_4_ could persist in sediments until May 2011. CH_4_ accumulation may result from long-term anaerobic hydrocarbon degradation (Widdel and Rabus, 2001), which would be in agreement with the presence of *Desulfobacterium* (Liu and Liu, 2013). Alternatively, acetotrophic methanogens may have been stimulated by an increase in acetate produced by acetogenic hydrocarbon-degrading SO_4_-reducing bacteria (SRB). Together with the upregulated nitrification in September–August 2010 (Mason et al., 2014), the available data suggest that persistent oil-contamination affects O_2_ seafloor levels indefinitely, supporting anaerobic benthic microbial activity.

## *Ex Situ* Microbiology and Lab-Scale Hp Experiments

None of the *ex situ* experiments on DWH deep-sea samples applied HP (Hazen et al., 2010; Valentine et al., 2010; Bælum et al., 2012; Redmond and Valentine, 2012; Gutierrez et al., 2013b; McKay et al., 2013; Mason et al., 2014; Yergeau et al., 2015; Dombrowski et al., 2016). Despite the persistence of *n*-alkanes and mild beta-oxidation upregulation, *ex situ*^14^C-experiments reported high *n*-alkane degradation in sediment (Mason et al., 2014) and water samples (Yergeau et al., 2015). Oiled beach sands were enriched in *Gammaproteobacteria* (*Alcanivorax*, *Marinobacter*) and *Alphaproteobacteria* (*Rhodobacteraceae*; Kostka et al., 2011). While some *Rhodobacteraceae* were found in deep-sea waters (Dubinsky et al., 2013) and sediments (Mason et al., 2014), neither *Marinobacter* nor *Alcanivorax* were enriched, contrary to other *Alteromonadales* and *Oceanospirillales* (Hazen et al., 2010; Dubinsky et al., 2013; Yang et al., 2014). Other hydrocarbonoclastic *Oceanospirillales* as *Thalassolituus, Oleiphilus, Neptunomonas*, or *Oleispira* were only reported because they consist of species closely related to the *Oceanospirillales* group identified in the plume (97%, *Oleispira antarctica* and *T. oleivorans*; Hazen et al., 2010). These isolates degrade long-chain hydrocarbons (Joye et al., 2014), which were not particularly enriched in the plume (**Table 1**). Low temperature was proposed to account for this (Redmond and Valentine, 2012), although species as *O. antarctica* are psychrophilic (Yakimov et al., 2003) and many of these genera populate hydrocarbon-seeps in the Gulf of Mexico (King et al., 2013).

The reason why these predominant hydrocarbonoclastic genera were not enriched at deep-sea is currently unknown. A moderately piezophilic *Marinobacter hydrocarbonoclasticus* strain could grow on C_16_ at 35 MPa (∼3 times higher than DWH plume HP; Grossi et al., 2010). As *Alcanivorax* abundance in bathypelagic water (Gutierrez et al., 2013b) and sediments was low (Kimes et al., 2013) and unrelated to hydrocarbons (Kimes et al., 2013) its contribution to deep-sea bioremediation was considered negligible (Gutierrez et al., 2013b). *Alcanivorax* isolates were obtained from decompressed water samples (Gutierrez et al., 2013b) resembling results and HP conditions for oil mousses (Liu and Liu, 2013) and beach sands (Kostka et al., 2011). *Alcanivorax* species isolated from 2682 to 5000 m bsl (up to 50 MPa, Liu and Shao, 2005; Lai et al., 2011) could not grow below 10°C, i.e., at much higher temperature values than those registered for these depths (<4°C). Another *Alcanivorax* strain was isolated from 668 m bsl (∼6.7 MPa, Lai et al., 2013). However, the isolation protocols employed in these studies did not apply HP. The first HP experiments on *Alcanivorax* were reported by the present group (Scoma and Boon, 2016; Scoma et al., 2016a,b). A mild increase to 5 MPa (∼500 m bsl) was sufficient to impair cell replication in *Alcanivorax dieselolei* and *A. jadensis*. Increase to 10 MPa in *A. dieselolei* (approximately the oil plume HP) further impaired growth, in concomitance with a general downregulation of its genome expression. The few upregulated pathways related to protein translation, energy production and Na^+^ transporters (Scoma et al., 2016a,b). Similarly, in the type strain *Alcanivorax borkumensis* SK2 the increased cell damage at 10 MPa was consistent with the intracellular accumulation of the piezolyte ectoine, and further studies on hypo- and hyper-osmotic stimulation highlighted that enhanced cell metabolism or integrity did not improve growth at 10 MPa (Scoma and Boon, 2016).

Hydrostatic pressure affects enzyme folding (Oger and Jebbar, 2010), cellular components (Bartlett et al., 1995) and functions, which may be gradually downregulated, triggered (Clouston and Wills, 1970; Kalchayanand et al., 2002; Ishii et al., 2004) or non-linearly induced (Pagàn and Mackey, 2000). *Sphingobium yanoikuyae* growth was suddenly impaired at >8.8 MPa when supplying naphthalene (or glucose, Schedler et al., 2014). *A. borkumensis* cultures growing on C_12_ were inactivated at 5 MPa but could grow at 10 MPa (Scoma et al., 2016b). Both growth and C_16_ degradation rates were reduced at 15 MPa in *Rhodococcus qingshengii* (Schedler et al., 2014). HP impact on oil biodegradation rates has been critically overlooked. Hazen et al. (2010) proposed half-lives of 6 days for plume-related *n*-alkanes supported by *ex situ* ambient pressure experiments, while *in situ* measurements indicated 1 month half-lives for water-soluble petroleum-hydrocarbons (Reddy et al., 2012). The main constraints to deep-sea oil bioremediation are yet to be elucidated. Injection of 3 × 10^6^ liters of COREXIT dispersant at deep sea implies that bioavailability was considered a major issue. The negligible degradation rates of its key components and its unknown effect on deep-sea environments (Kujawinski et al., 2011) challenge this assumption. Understanding whether biosurfactant production or microbial adhesion to hydrocarbons is limited by HP and/or temperature may already assist policymakers in establishing efficient protocols for deep-sea bioremediation.

## Future Perspectives

The limited literature on lab-scale oil degradation under HP (Schwarz et al., 1974, 1975; Grossi et al., 2010; Schedler et al., 2014; Scoma and Boon, 2016; Scoma et al., 2016a,b) does not explain how microbes cooperate/compete for petroleum-hydrocarbons, extracellular metabolites, O_2_ or nutrients.

The main structure-function mechanisms shaping microbial communities following deep-sea spills are unclear. Cell growth in the plume was proposed as a main response to oil release (Hazen et al., 2010; Mason et al., 2012; Kleindienst et al., 2015). However, metadata also indicated an enhanced response to stress (Hazen et al., 2010; Rivers et al., 2013) including starvation (Lu et al., 2012; Rivers et al., 2013), carbon storage (Rivers et al., 2013), and resistance to metals (Hazen et al., 2010; Lu et al., 2012). Similar results were obtained with sediments (Kimes et al., 2013; Mason et al., 2014; Yergeau et al., 2015). The *M. hydrocarbonoclasticus* tested at 35 MPa by Grossi et al. (2010) used C_16_ to feed both growth and the accumulation of intracellular wax esters. However, microbial dynamics may respond to compounds not typically analyzed in field samples as polar compounds (Reddy et al., 2012).

State-of-the-art technology can already be used to face the problem of deep-sea oil bioremediation by integrating molecular, physiological, and biochemical tools in a fully controlled environment. *In situ* hydrocarbon degradation rates must be confirmed by *ex situ* HP experiments *in vivo* and *in vitro*. Recent reports on methanotroph enrichments stressed the importance of cultivation techniques (Dedysh et al., 2012), supporting the use of synthetic communities to highlight the contribution of different individual genera to biodegradation activities. Some microbial representatives produce EPS to increase oil bioavailability for the benefit of the whole community (Gutierrez et al., 2013a). Enriched communities may be composed of opportunistic microbes which do not contribute to oil remediation. Defining the exact role of primary oil degraders with respect to other genera is key to addressing the specific requirements of each representative, and may explain how deep-sea areas are evolving following oil spills. Continuously operated HP systems may prevent accumulation of toxic compounds and provide more accurate data on microbial kinetics (Zhang et al., 2010, 2011). Isotopic experiments under HP should clarify whether oil fuels cell division, production of secondary metabolites or other unexpected activities. Improved biodegradation rates through the supply of critical nutrients should be compared with the impact of dispersants. The fate of tar components should be characterized, together with the role of SRB in long-term exposed sediments. These considerations must extend to temperature, as warmer seas may possess a different microbial potential (e.g., the warm bathypelagic Mediterranean sea). Deep-sea HP and low temperature impact the physicochemical state of the oil (Reddy et al., 2012) affecting its bioavailability. Low temperature is expected to slow microbial kinetics and select for psychrophiles (Atlas and Bartha, 1972). Provided that deep-sea oil bioremediation is affected by the interplay between several biological and physical factors, laboratory experiments must consider HP and temperature simultaneously to mimic deep-sea conditions.

## Author Contributions

AS conceived the project and wrote the review. MY and NB discussed and supervised the project and co-wrote the review.

## Conflict of Interest Statement

The authors declare that the research was conducted in the absence of any commercial or financial relationships that could be construed as a potential conflict of interest.
